# Device design methodology and formulation of a protein therapeutic for sustained release intraocular delivery

**DOI:** 10.1002/btm2.10121

**Published:** 2018-12-03

**Authors:** Erica B. Schlesinger, Daniel A. Bernards, Hunter H. Chen, James Feindt, Jingtai Cao, Daniel Dix, Carmelo Romano, Robert B. Bhisitkul, Tejal A. Desai

**Affiliations:** ^1^ Graduate Program in Bioengineering University of California San Francisco CA 94158; ^2^ Formulation Development Group Regeneron Pharmaceuticals Tarrytown NY 10591; ^3^ Dept. of Bioengineering and Therapeutic Sciences University of California San Francisco CA 94158; ^4^ Ophthalmology Research Regeneron Pharmaceuticals Tarrytown NY 10591; ^5^ Dept. of Ophthalmology University of California San Francisco CA 94158

**Keywords:** drug delivery, intravitreal device, polycaprolactone, protein formulation, sustained delivery, thin film device

## Abstract

Despite years of effort, sustained delivery of protein therapeutics remains an unmet need due to three primary challenges – dose, duration, and stability. The work presented here provides a design methodology for polycaprolactone reservoir‐based thin film devices suitable for long‐acting protein delivery to the back of the eye. First, the challenge of formulating highly concentrated protein in a device reservoir was addressed by improving stability with solubility‐reducing excipients. Next, predictive correlations between design parameters and device performance were developed to provide a methodology to achieve a target product profile. Prototype devices were designed using this methodology to achieve desired device size, release rate, therapeutic payload, and protein stability, assessed by *in vitro* studies. Finally, prototype tolerability was established in a non‐human primate model. The design methodology presented here is widely applicable to reservoir‐based sustained delivery devices for proteins and provides a general device design framework.

## 1 INTRODUCTION

Treating back of the eye (BotE) disease with protein therapeutics is challenging due to the eye's protective biological barriers, which require direct administration of protein into the vitreous.^1^, ^2^ As biologic protein drugs, such as ranibizumab and aflibercept, continue to demonstrate their effectiveness and gain prominence for treating BotE disease,^3^ regimens to individualize the frequency of injection based on the patient specific disease activity have also become common practice. The burden of administration for both clinician and patient has led to the emergence of as needed (PRN) treatment strategies in attempt to reduce injection frequency. However, such reactive regimens only trigger re‐treatment when patients' conditions worsen, and the consequence is insufficiently frequent and irregular treatment that can lead to suboptimal clinical outcomes.^4^, ^5^, ^6^, ^7^ By enabling predictable dosing to aid compliance and by ensuring continuous intraocular therapeutic levels, a device for sustained protein delivery for BotE would not only reduce the treatment burden, but would also have the potential to maximize and maintain vision benefits for patients with retinal diseases in clinical practice with these widely used drugs. Specifically, a sustained release device requiring dosing less than four times a year, as opposed to 6–12 times per year as in current practice, would meaningfully improve patient care.

Despite years of efforts and advances in research, preclinical, and clinical development, sustained delivery systems for protein therapeutics remain an unmet need^1^, ^2^, ^3^: among ocular drug delivery technologies that have reached clinical trials, the Port Delivery System is the only technology to accommodate a protein therapeutic.^8^ Dose, duration, and stability are the three primary challenges in sustained protein release, particularly for intraocular delivery.^3^, ^9^ Protein therapies for BotE treatment require repeated bolus doses as frequent as 0.5 mg per month (ranibizumab) or 2 mg every 2 months (aflibercept).^10^, ^11^ To limit the administration of a BotE device to less than 4 times per year, delivery systems will require drug payloads of at least several milligrams, not including necessary stabilizing excipients. To achieve such high payloads in a size suitable for intravitreal administration, protein concentration will be many fold greater within a reservoir device than that found in existing intravitreal liquid formulations. Unfortunately, proteins at such high concentrations are prone to aggregation under physiological conditions.^12^ Accordingly, to achieve a relevant dose and duration, a new formulation approach is needed that is suitable for a long‐acting implantable reservoir device.

In addition to the form and formulation challenges of the delivery system itself, there are many hurdles to translate early research on such a system into the clinic. Throughout the development process, reservoir devices will necessarily change and evolve to meet the specifications set forth in the target product profile and clinical trial design. Hence it is paramount to have a thorough understanding of how device design parameters influence and govern device performance. This understanding allows for the scale‐up and scale‐down of device dimensions as required during the different phases of development, for increasing or decreasing dosing as necessary to accommodate preclinical and clinical studies, and for accommodating changes to protein formulation as the system is optimized for stability. The purpose of this work is to provide a design methodology for polycaprolactone (PCL) reservoir‐based thin film devices^13^, ^14^ suitable for long acting delivery to the BotE, which enables design of devices with a particular size and shape that achieve a desired release rate, duration, and protein stability to achieve sustained therapeutic efficacy.

First, the challenge of formulating highly concentrated protein in a device reservoir is presented along with an approach to improve stability utilizing solubility‐reducing excipients. Next, predictive correlations between design parameters and device performance are developed to provide a straightforward methodology to achieve a specified device behavior. Prototype devices are designed using this methodology to achieve desired specifications in terms of device size, release rate, therapeutic payload, and long‐term protein stability. Finally, tolerability of prototype devices is established in a non‐human primate model and is a first major step toward subsequent pharmacokinetics (PK) and efficacy studies. The experiments and devices presented in this article use a solid form of the recombinant protein aflibercept, which is a vascular endothelial growth factor inhibitor used in the treatment of ocular diseases. While these studies focus on aflibercept as a model protein, the fundamental principles and underlying approaches presented are generically applicable and could be applied to any protein therapeutic.

## 2 RESULTS

### 2.1 General device design

**Figure 1:**
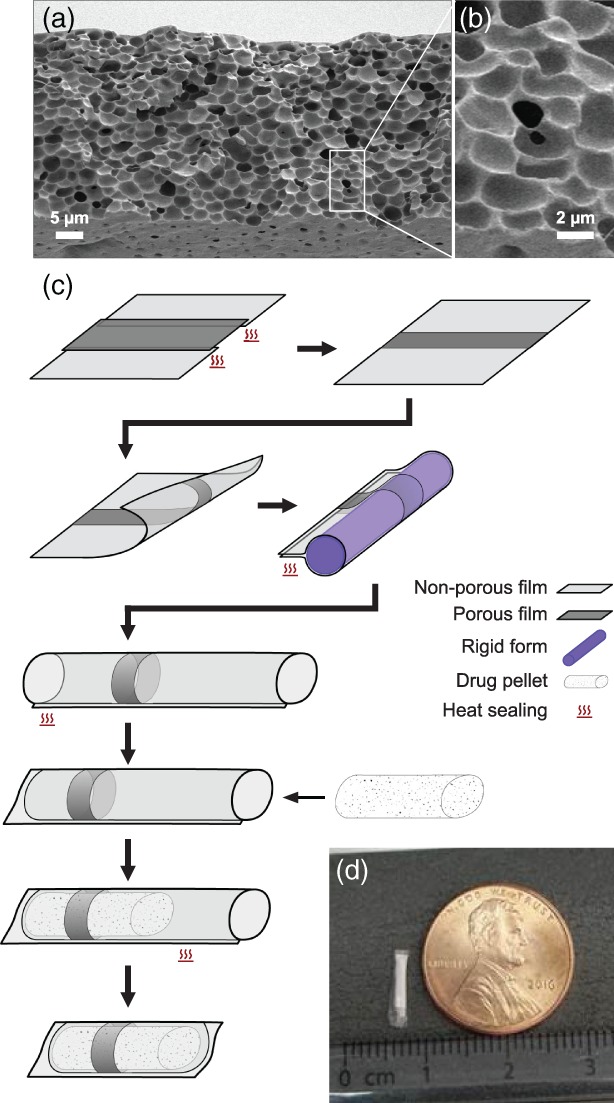
Fabrication of thin film device. (a) A cross‐sectional scanning electron microscope image of a typical mpPCL film, with (b) a magnified example of the interpore connectivity. (c) Devices are fabricated by combining porous (dark gray) and nonporous (light gray) films and sealing together with heat. The resulting film with a strip of mpPCL is formed around a cylindrical mold (purple) and heat sealed along the length of the mold. Prior to loading the protein pellet (white speckled), one end is heat sealed, and once loaded, the open end is sealed. Finally, the device is trimmed to remove excess polymer. (d) Photograph of a example lab‐scale device

**Figure 2:**
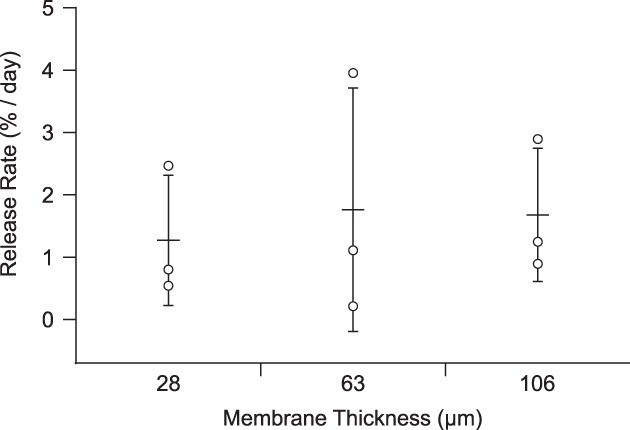
Dependence of release rate on membrane thickness. Release rate plotted for three membrane thicknesses in the range of 28–106 μm (*N* = 3 for each thickness; points correspond to individual devices, and error bars represent ± SD relative to the mean). Means comparison indicated no statistically significant difference across thicknesses for α = 0.05

**Figure 3:**
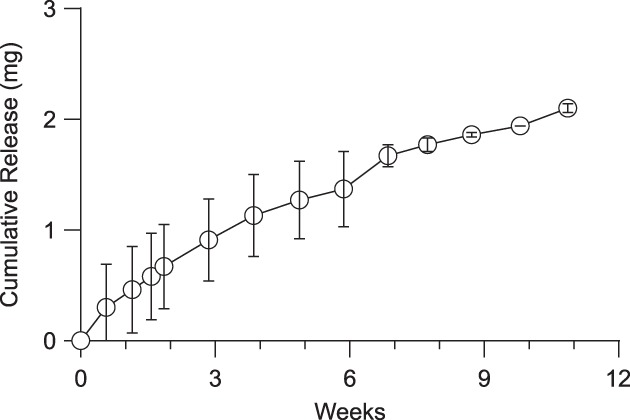
Sustained release of research‐grade aflibercept from thin film device. Cumulative release of aflibercept from devices over the course of 11 weeks in BSS‐like release media at 37 °C. (*N* = 3, error bars represent ± SD)

Figure 1a shows a cross‐sectional scanning electron microscopy (SEM) image of a prototypical microporous PCL (mpPCL) film used in the fabrication of sustained release devices. An example of the connectivity between micron‐sized pores (two visible and distinct pores with an interconnecting channel) is shown in Figure 1b. While many interconnecting channels can be observed upon close inspection, the majority of instances include a pore in the bulk of the film, where it is technically challenging to demonstrate connectivity. The submicron connections between pores are hypothesized to be the rate limiting step to protein permeability rather than the tortuous diffusion through the microscale pores. This is further supported by the observation that release kinetics compared across devices with varied membrane thickness ranging from 28 to 106 μm were not significantly different across membrane thickness (*p* = .9043; Figure 2). The resulting sustained release profile yields kinetics that fall between zero and first order (Figure 3). in vitro release kinetics, insensitivity to membrane thickness, and nanometer scale of the rate limiting pore‐size suggest aflibercept transport across these membranes was dominated by Knudsen diffusion.^15^, ^16^

To construct devices, a combination of microporous PCL (mpPCL) and nonporous PCL (npPCL) films were used. Rather than altering membrane properties to tune release rates, mpPCL membrane area was adjusted to provide desired release rates. These hybrid membranes were fabricated by heat sealing npPCL and mpPCL films to achieve the desired porous area (Figure 1c). As release was insensitive to membrane thickness, devices were fabricated from films approximately 25–35 μm thick, which balanced minimizing film thickness with robustness when handled. A series of subsequent heat sealing steps yielded cylindrical style devices that were loaded with a solid formulation of research grade aflibercept and excipients. The payload was compressed into a pellet and loaded into the device immediately prior to the terminal sealing step. Figure 1d shows a representative loaded device.

### 2.2 Protein formulation and stability

Protein within a sustained release device should remain stable at 37 °C throughout the duration of administration. While there are many mechanisms of instability for protein therapeutics, one of the greatest challenges is protein aggregation.^17^, ^18^, ^19^, ^20^ In general, dimers of some proteins may retain activity with a reduced efficacy, yet higher molecular weight species often have little or no efficacy.^21^, ^22^ Proteins are susceptible to aggregation when in solution, particularly at high concentrations. This can either be due to noncovalent hydrophobic interactions between protein molecules or covalent interactions between intermolecular residues, such as cross‐linking of disulfide bonds.^12^, ^20^ For a reservoir device loaded with a 3–6 month payload of protein (an estimated 1.5 mg minimum), the reservoir protein concentration will need to be at least an estimated 190 mg/ml (for a 1 x 10 mm^2^ cylindrical device), if not substantially higher. Both high concentration and the elevated physiological temperature increase the frequency of protein–protein interaction as well as the propensity for protein unfolding and resulting exposure of hydrophobic regions**.** 18 Consequently, there is typically a direct correlation between the concentration and temperature of a protein solution and its physical stability.^18^, ^23^, ^24^, ^25^, ^26^

Liquid protein formulations usually require refrigerated storage (2–8 °C) and contain excipients, such as sucrose or trehalose that act as thermal stabilizers to reduce the formation of dimers and high‐molecular weight species, particularly at elevated temperatures. Such strategies are effective in liquid formulations, but an implantable reservoir device must reside in physiological conditions (37 °C) for the duration of delivery, placing greater thermal stress on the protein. Furthermore, for an intraocular device, device volume is constrained and necessitates highly concentrated protein within the device (>175 mg/ml). Unfortunately, many common stabilizing excipients are also a poor match for reservoir‐based devices: sugars and other small molecule stabilizers are expected to rapidly diffuse out and deplete from a device reservoir given their small size compared to a conventional protein therapeutic. Consequently, an alternate formulation approach could improve protein stability for sustained delivery from a reservoir‐based device.

Protein mobility and aggregation are reduced in solid forms compared to solutions,^27^ and protein aggregation is known to generally increase with protein concentration.^18^ Thus, it was hypothesized that protein stability could be improved by sequestering the majority of protein in the solid state and limiting the maximum soluble concentration of protein within the reservoir. Adapting techniques to precipitate proteins for x‐ray crystallography, excipients such as PEG or dextran can be used to limit protein solubility and maintain a portion of protein in a solid state. Mechanistically, the reduction in solubility is attributed to an excluded volume effect (i.e., PEG prevents protein access to the solvent). At sufficiently high PEG and protein concentrations, protein will reach its solubility limit locally and be forced into the solid state.^28^ Generally, a reservoir concentration of less than 10 mg/ml is expected to sufficiently reduce aggregation and yield a viable formulation for reservoir devices.

**Figure 4:**
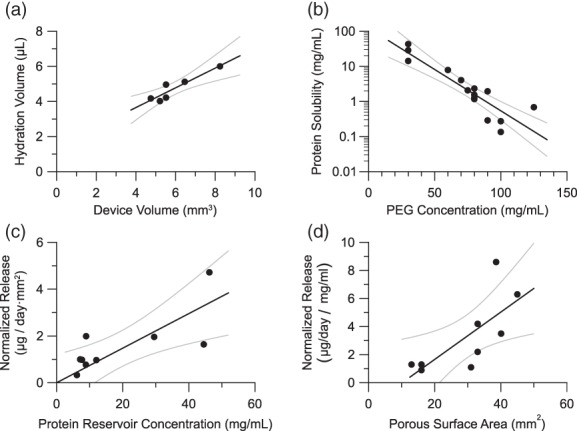
Correlations for device design. (a) Dependence of the hydration volume on the theoretical volume of a device reservoir. (b) Dependence of the solubility of aflibercept on the concentration of PEG in a solution. (c) Dependence of the aflibercept release rate on the reservoir concentration of aflibercept. Release rate is normalized by porous area. (d) Dependence of the aflibercept release rate on the porous surface area of the device. Release rate is normalized by aflibercept reservoir concentration. Gray lines indicate the 95% confidence interval on the linear regression. Pearson correlation coefficients are 0.89, 0.93, 0.75, and 0.73 for plots a, b, c, and d, respectively

To this end, medical‐grade polyethylene glycol (PEG; *M*_w_ = 3,350 Da) was explored as a possible solubility‐reducing excipient.^28^ Aflibercept solubility was measured with varied PEG concentration (Figure 4b), and a clear correlation was established between aflibercept solubility and PEG concentration. From this relationship, the PEG concentration should be at least 50 mg/ml to achieve an aflibercept solubility of less than 10 mg/ml. As devices were loaded with solid formulations, it was necessary to understand how device size corresponds to hydrated reservoir volume such that an appropriate mass of PEG was loaded. To assess hydration, aflibercept loaded devices were placed in buffer for 12–24 hr, and the mass change was attributed to water hydrating the device. Figure 4a shows the measured correlation between device volume and hydrated volume within devices. Based on this correlation, the mass of PEG required for a specific device volume can be determined to ensure the target protein solubility is achieved.

**Figure 5:**
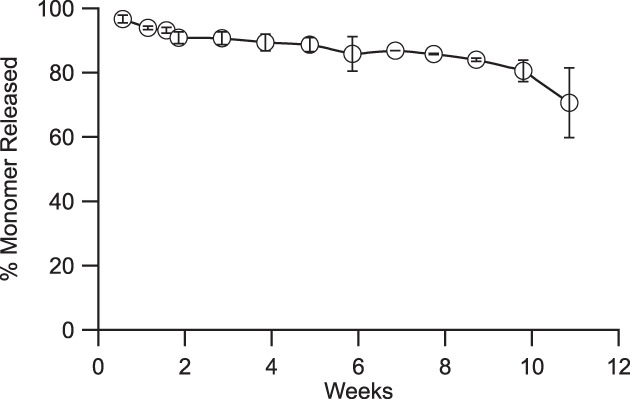
Released aflibercept stability. Percentage of total protein released that is monomer as measured over 11 weeks at 37 °C. (*N* = 5, error bars represent ±SD) reservoir devices were formulated with research grade solid aflibercept, buffer excipients, sucrose, and PEG

Utilizing this strategy, reservoir devices containing solid aflibercept formulations with PEG were assessed under in vitro conditions (Figure 5). When PEG was employed to achieve an estimated protein solubility of less than 10 mg/ml in the device reservoir, stability was significantly improved.^29^ In summary, with the PEG formulation, the rate of formation of dimer and high molecular weight species was reduced by 83% over a 1 month period relative to a reservoir formulation without PEG. Furthermore, the relative amount of total dimer and other high molecular weight forms after 9.5 weeks in the device reservoir containing the PEG formulation was equivalent to what was observed without the PEG formulation after only 11 days. This underscores the importance of controlling protein concentration in the reservoir and the viability of the proposed formulation technique. Although lowering protein solubility was shown to improve stability, there is a trade‐off between stability and achieving necessary protein release rates.

### 2.3 Controlling release rate

Protein concentration is the driving force for diffusion from the device reservoir, so the rate of protein release from the device is proportional to protein solubility in the reservoir. Normalized to porous area, Figure 4c shows the relationship between aflibercept release rate and aflibercept reservoir concentration (as determined from Figure 4b). In the overall system design, it is undesirable to limit the protein solubility completely, and aflibercept reservoir solubility should exceed a lower limit of 2 mg/ml to allow sufficient release in this system. Below this limit, aflibercept release became too slow to achieve and maintain an efficacious tissue concentration and is, therefore, unsuitable for devices. Intuitively, adjusting the porous surface area scaled directly with aflibercept release. Figure 4d shows the dependence of release rate on the porous surface area when normalized to account for variation in reservoir aflibercept concentration.

### 2.4 Design methodology

**Figure 6:**
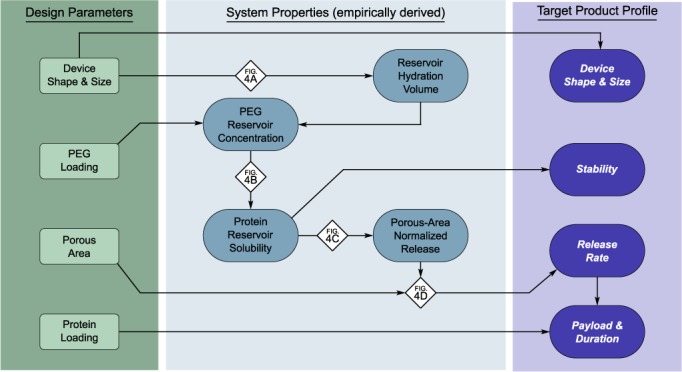
Design methodology for protein release from thin film devices. Schematic diagram shows how design parameters influence the target product profile via various system properties. Referencing the established correlations (diamonds), devices can be designed in a systematic way to achieve a desired target product profile

A clinically relevant, efficacious device must satisfy a target product profile that attains critical requirements, including release rate, protein payload, protein stability, and device size. Certain parameters are controlled directly, such as device size or protein payload; others are controlled indirectly, such as how PEG loading impacts protein stability and release rate. Figure 6 lays out a design methodology showing how design parameters control system properties that in turn allow one to obtain a desired target product profile. The empirically derived relationships shown in Figure 4 are employed at the nodes shown on Figure 6 to satisfy the requirements of the target product profile. The empirical relationships shown in Figure 4 are not necessarily generalizable to an arbitrary protein and would likely need to be determined for the specific protein of interest.

Briefly, a device size is selected, and the resulting reservoir hydration volume can be determined from Figure 4a. As protein stability is predicated by the protein reservoir concentration, an acceptable protein reservoir concertation is selected to achieve stability requirements. This protein concentration is used to determine the PEG reservoir concentration from Figure 4b, and the mass of PEG loaded into the device reservoir can be calculated using the reservoir concentration along with the hydration volume. Given the predetermined reservoir concentration, an area‐normalized release rate can be predicted from Figure 4c, and the porous area of the device can be selected to achieve the desired release rate. Alternatively, both porous area and protein reservoir concentration both impact release rate, so an iterative approach may be employed to optimize release rate and protein stability while maintaining attainable PEG loading and porous area. In addition, one must also verify that the device volume is physically large enough to accommodate the total payload of protein and PEG.

### 2.5 Prototype preclinical devices

Prototype preclinical devices were designed to achieve the following target product profile: devices were less than 1.5 mm in diameter and 10 mm in length, were loaded with at least 3 mg aflibercept, and were capable of a near linear release rate of 1–50 μg/day. Three prototype devices were designed, fabricated, and characterized by in vitro evaluation of aflibercept release and corresponding stability, as measured via size‐exclusion ultra‐performance liquid chromatography (SE‐UPLC). Device design parameters were determined using the methodology described in Figure 6 and with the empirical relationships shown in Figure 4. Using this design methodology, prototype devices were fabricated and tested under simulated physiological conditions. Appendix Table A1 provides a summary of the prototype devices. Figure 3 illustrates the near zero‐order release profile from these devices with a steady‐state release rate of 25 μg/day through 11 weeks (Figure 3).

### 2.6 In vivo tolerability

To evaluate ocular tolerance, placebo and active sustained‐release devices were implanted bilaterally in African green monkeys. Active devices contained a formulation of solid aflibercept, sucrose, and PEG, and placebo devices contained only sucrose and PEG. The active and placebo groups were both included in this study design to differentiate between ocular tolerability in relation to the device (size, geometry, material properties, etc.) and this novel solid aflibercept formulation. Active and placebo devices were 8.1 ± 0.6 mm and 7.9 ± 0.8 mm long, respectively. Diameter was not directly measured to minimize unnecessary handling, but previously fabricated devices that were nominally equivalent had diameters of approximately 1 mm. Devices were implanted on Day 0 from the superior temporal quadrant in the right eye and from the inferior temporal quadrant in the left eye. The differing locations for each eye were selected to allow better exposure of the implantation site. Following device placement, the incision was sutured, and subsequent visual inspection did not reveal persistent vitreous leakage or implantation‐associated ocular injury, such as intraocular hemorrhage or retinal detachment.

**Figure 7:**
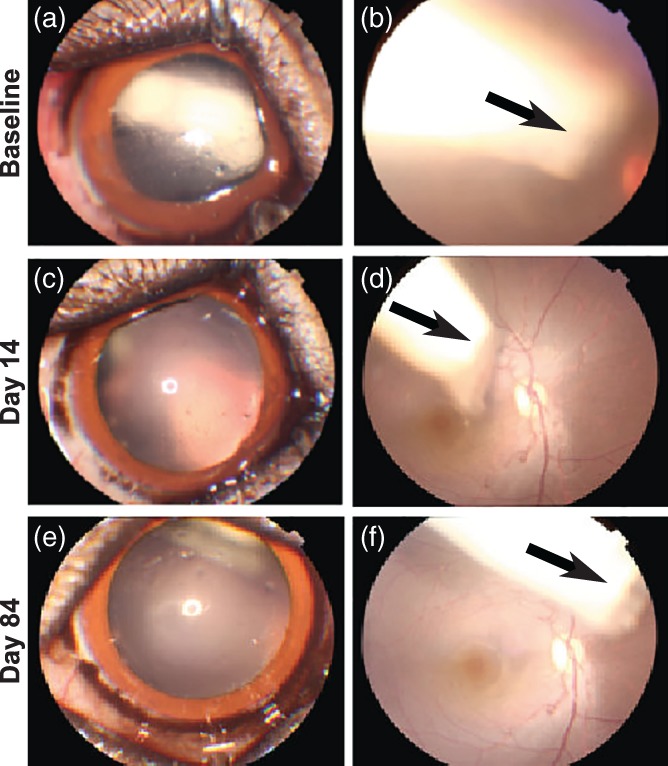
Anterior segment and fundus photographs. Representative anterior segment and color fundus photographs obtained from an eye receiving device with placebo (a,b) immediately post‐implantation, (c,d) on day 14, and (e,f) day 84. Pupil was dilated to facilitate visualizing and imaging device. The device moved from the anterior vitreous to the posterior vitreous over the time (arrows). No signs of retinal hemorrhage, exudates, edema, detachment and vascular congestion, or tortuosity were observed

Devices were left resident for 84 days, and fundus photography and post‐ophthalmic examinations were performed at regular intervals. Fundus photographs were taken to assess device appearance and location and to evaluate signs of adverse reaction. In general, devices were positioned in the anterior vitreous following insertion and shifted toward the posterior vitreous with subsequent observation (Figure 7). Appearance of the two treatment groups lacked significant differences as observed between early and later examination time points. Neither fundus photography nor retinoscopy revealed signs of retinal trauma (hemorrhage, exudates, edema, or detachment), vascular congestion, or tortuosity (Figure 7). While eight placebo and eight active devices were implanted, one animal in the active device group died on Day 6 due to unrelated complications, so the active device group had an *N* = 6 eyes rather than *N* = 8 eyes as in the placebo device group.

Ophthalmic examinations were conducted periodically to evaluate inflammatory response post‐implantation. Mild intraocular inflammation in the anterior segment was observed in the first week following implantation for both active and placebo devices: inflammation response was comprised of mild aqueous flare, aqueous cells, fibrin clots deposited on the lens capsule, and iris hyperemia. Anterior segment inflammation self‐resolved within 2–4 weeks post‐implantation for both groups. In the posterior segment of the eye, transient vitreous cells were observed primarily between Days 6 and 28 in the placebo group, and throughout the study in the active group.

**Figure 8:**
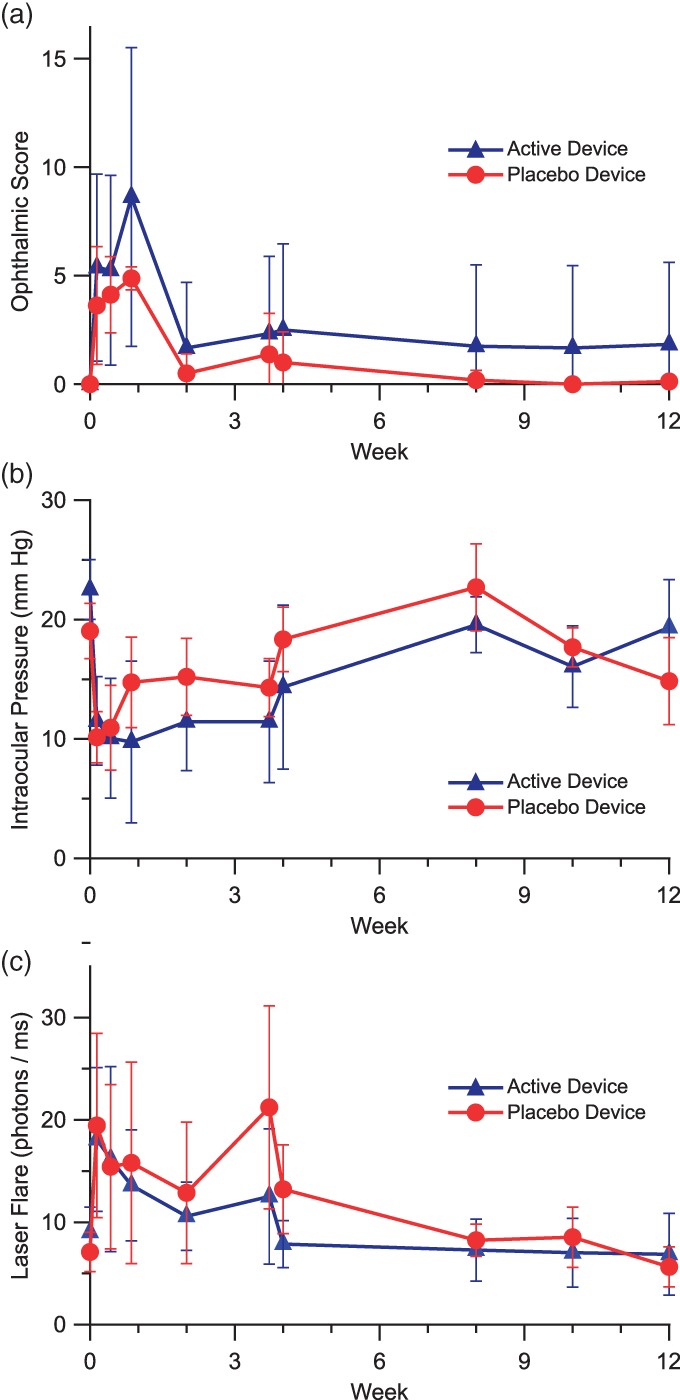
Non‐human primate response to thin film devices. Quantitative results of ophthalmic examinations following intravitreal implantation of devices in African green monkeys over the course of 12 weeks. Devices loaded either with sucrose and PEG (placebo; *N* = 8 eyes) or with solid aflibercept, sucrose, and PEG (active; *N* = 6 eyes). (a) Modified McDonald‐Shadduck score (±95%CI). (b) Intraocular pressure (±SEM). (c) Laser flare photometry measures (±SEM)

To quantify the overall inflammatory response, a modified McDonald‐Shadduck scoring system was used (Figure 8a).^30^ Initial mild inflammation during the first week post‐implantation resolves by week two. Nonparametric analysis of the pathology score indicated a statistically significant difference between these two treatment groups (Median test, Chi‐square = 74.3277, *p* < .0001). The acute inflammatory response is likely induced by the trauma of the implantation process rather the device itself. These results indicate suitable ocular tolerability of both the placebo and active device.

Intraocular pressure was monitored during the study observation period (Figure 8b). In comparison with the baseline level, mean IOP was significantly lower only at Days 1 and 3 in the placebo group, while persistently lower IOPs were measured through Day 26 in the aflibercept group. Data were analyzed by two‐way anova with repeated measures, in which Factor 1 (treatment group) *F* = 2.7587, *p* = .0992; Factor 2 (time point) *F* = 11.8263, *p* < .0001; “Factor 1 * Factor 2” *F* = 2.1699, *p* = .0284. A low IOP was observed typically in eyes exhibiting active intraocular inflammation in the anterior segment, reflecting decreased aqueous humor secretion. Loss of some vitreous during the implantation procedure may also have contributed to the lower IOP at early time points.

Laser flare photometry provides an objective quantification of aqueous flare, reflecting the amount of protein in aqueous humor. Laser flare measures were obtained at all time points in the majority of eyes, except for a few time points when there were significant quantities of fibrin in the anterior chamber or corneal surface dryness or abrasion. Compared to baseline levels, mean laser flare measures had a mild increase within 4 weeks post‐implantation, but the differences were not statistically significant in both groups except for Days 1 and 26 in the placebo group (Figure 8c). Data were analyzed by two‐way anova with repeated measures, in which Factor 1 (treatment group) *F* = 2.4397, *p* = .121; Factor 2 (time point) *F* = 5.8525, *p* < .0001; “Factor 1 * Factor 2” *F* = 0.7268, *p* = .6838. The notable increase in laser flare at Day 26 in the placebo group was driven by an increased measure in the eye of two different animals and was likely due to corneal surface dryness because no aqueous flare or cells were observed by slit‐lamp biomicroscopy at that time point.

**Figure 9:**
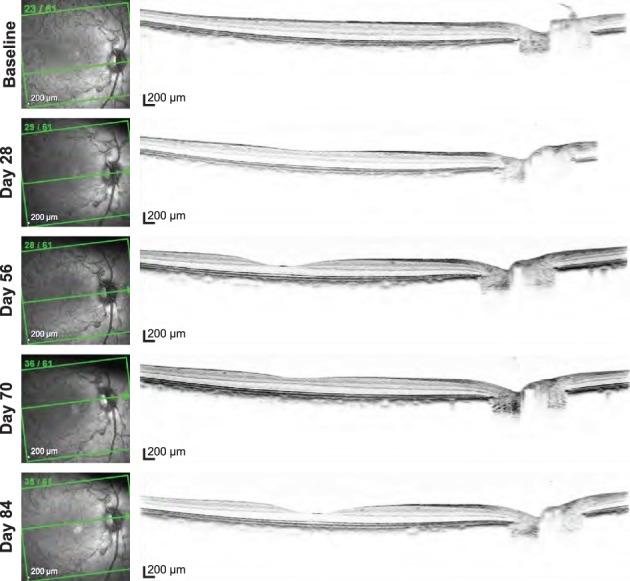
Representative OCT imaging. OCT images obtained from a placebo device at baseline screening, days 28, 56, 70, and 84 presented from top to bottom. No signs of retinal edema, detachment, or significant vitreous pathology were exhibited in the OCT images

Follow‐up ocular coherence tomography (OCT) examination was conducted at Day 28 and at each examination time point thereafter. The central cross sectional images are presented in Figure 9. No signs of retinal edema or detachment were exhibited in the OCT images with both the placebo and active device.

## 3 DISCUSSION

The challenge of developing a sustained release device requires shaping many interconnected parameters around a set of device goals and requirements to achieve the desired TPP. Many of these are mutually exclusive by nature, so it is important to have a well‐developed understanding of device performance and behavior. The device design approach presented here was implemented to allow for easy scaling and adjustment of device properties, which provides invaluable versatility during preclinical development. For instance, due to the challenges of fabricating and performing measurements on devices suitable for ocular implantation, early development benefits from prototypes larger than a commercial product to improve the ease of lab‐scale fabrication and reduce experimental measurement error. Furthermore, in the early stages of development, devices are made by hand, whereas subsequent production almost certainly necessitates a high degree of standardization and likely some level of automation. In the case of the reservoir‐based sustained release device presented here, the critical parameters for an effective device were identified as protein payload, sustained release rate, protein stability, and physical size.

One of the more challenging aspects of a reservoir device is protein stability within the reservoir given the high protein concentration. The inclusion of PEG in device reservoirs was employed to reduced solubility of the contained protein. The lower protein solubility improved stability, and the rate of formation of dimer and other high molecular weight species was decreased to an acceptable level to retain efficacy for the duration of drug release. The solubility effect associated with PEG depends on the concentration of PEG within the device reservoir and does not directly depend on protein loading; however, for a fixed device size there is a maximum protein loading based on required PEG concentration for stability and the densities of solid PEG and protein loaded into the device.

$$m_{\text{protein}}^{\max}=\rho_{\text{protein}}\cdot V_{\text{device}}\cdot \left(1-\frac{C_{\mathrm{PEG}}}{\rho_{\mathrm{PEG}}}\right),$$

where $m_{\text{protein}}^{\max}$ is the maximum protein loading, *ρ*_protein_ is the density of protein in the solid state, *V*_device_ is the device volume, *C*_PEG_ is the reservoir concentration of PEG, and ρ_PEG_ is the density of PEG. For any sustained release device, the purity of the protein therapeutic released at the end of the device lifetime is critical to ensuring effective therapy through the life of the device. Because steady‐state behavior in the eye of a protein delivered from a sustained release device is largely unexplored in vivo, the release rate of active protein required for a clinical effect can only be viewed as a rough estimate: this highlights the absolute need for a versatile device that can be engineered to suit the requirements of any specific therapy.

Critical to the design approach presented here is a set of descriptive empirical correlations between design parameters and system properties that enable design to a specific product profile. These relationships will be unique for any particular protein and PEG combination, and such correlations would need to be redeveloped for a novel combination. Still, one may expect the relationship between device size and hydration volume to be relatively insensitive to specific protein as it pertains primarily to the physical geometry of the device. Also, in the measurement of device hydration presented here, devices were allowed to fully hydrate and in the process some protein will be released. In this case, the slow release only yields approximately 15 μg of aflibercept in 12 hr whereas >4 mg of water enters a typical device upon hydration. Consequently, the mass of protein loss can be considered negligible when calculating the hydration volume. Excluding the protein solubility dependence, the correlations presented in Figure 4 make use of fine mass measurements that are susceptible to analytical instrument error, which can be seen in the data spread. As a result, when applied to the design methodology some variation from the correlative models can be expected. This was evident in the prototype devices that were expected to have a release rate of 30 μg/day rather than the observed rate of 25 μg/day.

For a sustained release device, a constant rate of release is preferred to provide a consistent level of therapeutic. While the prototype devices here did not exhibit perfect zero‐order release, a predictable rate of release can be obtained. The prototype devices introduced here achieved a release rate of 25 ug/day with a payload of roughly 3 mg. Thus, these devices are expected to deplete after approximately 120 days. To design for 6 month devices, the payload must be increased by at least 50%. Alternatively, if the release rate can be reduced and remain effective, devices may be redesigned to achieve a decreased release rate.

Previous studies in rabbits have established ocular tolerance of PCL thin films as well as several drug–device combinations.^14^, ^31^, ^32^, ^33^ However as with any combination product, it is the system dimensions, the material, and the drug that are integral to device tolerance. To assess ocular tolerance of these devices, studies were performed in African green monkeys. This work represents the first ocular example of such thin film sustained release devices in non‐human primates and was not designed to assess PK or efficacy of the devices. In the future, an enhanced manufacturing infrastructure and processes would enable a rigorous assessment of PK and efficacy. In general, clinical scores remained low, near or at baseline, and both placebo and active devices were well‐tolerated in vivo for up to 12 weeks, demonstrating acute and long‐term tolerability. The PCL composition used in this work did not reach the terminal stages of degradation within 12 weeks, and evaluating the effect of the soluble polymer degradation products generated during the final stages of device degradation was not an endpoint of this study. Additional device development and in vivo work will be required to evaluate the complete degradation of these devices in vivo.

For these tolerability studies, an initial mild inflammatory response was observed across devices. This inflammatory response naturally resolved within a few weeks and appeared to be associated with the incision and implantation procedure, rather than induced by the device. Although expected to produce a moderate acute inflammatory response, the surgical route for implantation was preferred in this preclinical model to show proof of concept prior to the development of a custom applicator. However, the device presented here is envisioned for intravitreal implantation via syringe, similar to the Ozurdex device that is implanted with a custom injector with an outer diameter equivalent to a 22‐gauge needle.^34^ As was seen in the case of Ozurdex,^35^, ^36^ an augmented or custom injector needle is likely preferred and perhaps necessary for this class of devices.

In addition to the development of a custom injector, the devices described here will need to be further scaled down as manufacturing is improved, but it is important to understand how current devices compare to well‐tolerated injections. Common clinical intraocular protein injections are 50 μl.^10^, ^11^ The prototype devices here had a volume of less than 10 μl, which is easily less than volumes introduced in the course of known safe intravitreal injections. For protein therapeutics, device payload is expected to limit the minimum achievable device size.

Device size is practically constrained to a length of ≤10 mm and a cross‐sectional geometry that minimizes tissue damage during insertion. Furthermore, given an ocular diameter of approximately 20 mm,^37^, ^38^ an implant in excess of 10 mm is likely to cross the central visual axis and inhibit vision. Hence a maximum device size of 8–10 mm was selected as a starting point for device design and tolerability. Physiological constraints on device volume define device loading and thus duration of efficacy. For a 4‐month duration of administration, a rod‐shaped device can accommodate approximately 2.1 mg of total loading (therapeutic and all excipients) and remain suitable for delivery via a 22‐gauge needle. Depending on the dose requirement and formulation composition, the current device form may be therapeutically relevant with appropriate miniaturization. Future investigations of PK and PD will be required to assess the release rate and corresponding device loading to guide device size and design using the methodology presented here. Alternative device geometries integrated with custom injectors could potentially increase loading while reducing tissue damage during insertion. Although additional work is required to develop and evaluate such systems, the design methodology shown above would apply. The devices described here are implanted untethered and may migrate within the vitreous, yet device migration was not associated with negative tolerability outcomes in this study. If device migration proves to be a concern in future studies, the device design is amenable to being anchored. Additionally, further investigation is required to assess the impact of multiple sequential device implants and the timescale for resorption of these or similar devices.

## 4 MATERIALS AND METHODS

### 4.1 PCL film fabrication and characterization

All chemicals were obtained from Sigma‐Aldrich (St. Louis, MO) unless noted otherwise. PCL used had a *M*_n_ of 80,000 Da, and PEG used had a *M*_n_ of 2,050 Da. All films were draw‐cast onto a glass surface using a multiple clearance square applicator (Paul N. Gardner Company, Inc., Pompano Beach, FL). Nonporous PCL (npPCL) films were cast from an 80 to 200 mg/ml solution of PCL in dichloromethane. npPCL films were first allowed to air dry and were then annealed with a heat gun to just past melting and then cooled to room temperature. Solution concentration and clearance of the draw‐casting rectangle were used to obtain films of varying thickness. Microporous PCL (mpPCL) films were cast from a 200 mg/ml PCL and 200 mg/ml PEG in 2,2,2‐trifluoroethanol. Films were allowed to air dry, and then deionized water was used to dissolve the PEG phase from the mpPCL film, creating a porous structure. Thickness of npPCL and mpPCL films was measured using a micrometer. Porosity of mpPCL films was estimated by comparing the mpPCL film density to the density of PCL according to the following equation:

$$\%\text{porosity}=\left(1-\frac{m_{\text{film}}}{A\cdot t\cdot \rho_{\mathrm{PCL}}}\right)\times 100\%$$

where *m*_film_ is the film mass, *A* is the area of film, *t* is the film thickness, and ρ_PCL_ is the known density of PCL (1.125 g/ml). Film mass was measured with an analytical balance, film area was measured with calipers, and film thickness was measured with a micrometer. All mpPCL films used were 25–35 μm thick with 45–65% porosity. In the initial stage of combining mpPCL and npPCL films, npPCL thickness was selected to be roughly the same mass of mpPCL per area example, for a 30 μm thick 50% porosity mpPCL film, a 15 μm np PCL film was used).

### 4.2 Device fabrication (npPCL/mpPCL combination devices)

Devices were fabricated as shown schematically in Figure 1 and loaded with solid‐state aflibercept and research‐grade reagents**.** Throughout device fabrication, resistive heat sealing was used to fuse PCL films together and provide permanent device seals: a 32 average wire gauge nickel‐chromium wire was embedded between two polydimethylsiloxane (Sylgard 184, Dow Corning, Auburn, MI) slabs, PCL pieces to be sealed were aligned with the wire, and a constant current was applied to resistively heat the wire until PCL pieces were fused. To fabricate devices, npPCL was first heat sealed on either side of a narrow strip of mpPCL to form a single film spatially consisting of npPCL/mpPCL/npPCL. The film was then rolled around a cylindrical mold with a diameter chosen based on target device dimensions, and a cylinder was formed by heat sealing along the length of the mold. Next, one end of the cylinder was heat sealed closed to create a hollow cylinder with one open end. Solid research grade aflibercept and excipients that had been compressed into a pellet manually using various renditions of die molds depending on the desired pellet size were loaded into the cylinder, and the open end was heat sealed to complete the device.

Devices were weighed before and after loading of the solid‐state protein and excipients. Total solid loading was calculated based on the difference in mass of the loaded versus empty device, and protein loading was determined from the ratio of protein to excipients in the solid‐state formulation. Porous dimensions were measured with a caliper prior to device loading. The porous region was sufficiently far from the location of the terminal seal such that porous area was unaffected by the subsequent final sealing step. Overall, device dimensions were measured with a caliper after device loading and the terminal sealing step. Devices were loaded with solid‐state research grade aflibercept, which includes buffer excipients and sucrose, and where applicable with medical grade crystalline PEG (PEG3350; *M*_n_ ~ 3,350 Da; provided by Regeneron Pharmaceuticals).

### 4.3 Device hydration volume

Device hydration volume was determined for in vitro studies from the change in mass upon device hydration, measured after 12–24 hr incubation in release media. Device hydration volume was thus a measurement of the amount of water contained in the hydrated device reservoir, assuming no significant change in mass due to the release of reservoir contents upon hydration.

### 4.4 In vitro release

Devices were fabricated and loaded with research grade solid aflibercept and excipients as described above. Release of aflibercept from devices was evaluated by fully submerging devices in 0.5–1 ml release media (a proprietary media comprised of sodium phosphate, magnesium chloride, potassium chloride and surfactant, similar to balanced salt solution [BSS] media for intraocular irrigation, pH = 7.2) and incubating at 37 °C in sealed polypropylene tubes or glass vials. Devices were transferred into a fresh aliquot of release media every 1–3 days for the first 2 weeks and weekly thereafter; release media from the prior period of incubation was retained for analysis. For each release media sample, aflibercept concentration and purity (the relative concentration of monomer, dimer, high‐molecular weight, and fragmented species) were determined via size exclusion high performance or ultraperformance liquid chromatography (SE‐HPLC/UPLC), and total mass of aflibercept released over each time interval was calculated from the concentration and release media volume. The SE‐HPLC/UPLC method utilized an Agilent 1200 Series HPLC or Waters Acquity H‐Class UPLC equipped with an ultraviolet (UV) detector. To present device release as a function of time, cumulative release was calculated by summing the total mass of aflibercept released through all previous time points. To determine percentage monomer, a reference sample of aflibercept was used to identify the elution time of monomeric aflibercept and subsequently calculate the percentage monomer. To account for device‐to‐device porosity variation, some release profiles were normalized to porous surface area, where cumulative release was divided by the porous surface area measured during device fabrication.

### 4.5 Aflibercept‐PEG solubility

Assessment of aflibercept solubility as a function of PEG concentration was conducted in glass vials. Concentrated PEG solutions were added to aflibercept solutions of known concentration to target a range of PEG concentrations (30–150 mg/ml) and aflibercept concentrations (5–45 mg/ml) in the final solutions. The PEG and aflibercept mixture was allowed to equilibrate for at least 60 min, and the solution was then centrifuged to separate any solid matter from the supernatant, which was reserved for analysis. The supernatant was filtered through a 0.22 μm PVDF membrane spin‐filter, and aflibercept concentration and purity were measured using reverse phase and size‐exclusion ultraperformance liquid chromatography.

### 4.6 Tolerability in non‐human primates

#### 4.6.1 Device fabrication and characterization

Devices for tolerability study were fabricated using previously described methods. Active devices were loaded with solid aflibercept and contained 1.5 mg aflibercept (±0.2 mg), 0.8 mg PEG (±0.4 mg), and 0.09 mg excipients (±0.01 mg) from the solid aflibercept formulation. Placebo devices contained 5 mg sucrose (±0.8 mg) and 0.3 mg PEG (± 0.5 mg). Sucrose loading in the placebo devices was chosen to produce a device with comparable physical dimensions to devices in the active group. Active devices were measured to be approximately 1 mm in diameter and 8.1 mm long (±0.6 mm). Placebo devices were measured to be approximately 1 mm in diameter and 7.9 mm long (±0.8 mm).

#### 4.6.2 Enrollment and group assignment

Eight adult African green (*Chlorocebus sabaeus*) monkeys (six male and two female) were enrolled in the study and randomized to two treatment groups based on the baseline body weight and balanced with respect to sex. The six adult male African green monkeys ranged in weight from 3.25 to 6.52 kg, and the two adult females ranged in weight from 2.89 to 2.92 kg. Baseline ophthalmic and clinical exams were performed to confirm good health and suitability for study enrollment.

#### 4.6.3 Care and handling

All animals were anesthetized with ketamine/xylazine intramuscularly (8.0 mg/kg ketamine and 1.6 mg/kg xylazine in a sterilely mixed cocktail) for all procedures and ophthalmic evaluations. General well being was assessed before, during, and after sedation.

#### 4.6.4 Implantation of device

On study day 0, each monkey received intravitreal implantation of either an active device in each eye or a placebo device in each eye. Prior to implantation, the skin was shaved around the orbit, eyelashes were cut off, and pupils were dilated using topical 10% phenylephrine hydrochloride and 1% cyclopentolate hydrochloride ophthalmic solutions. Topical anesthesia (0.5% proparacaine) was administered in addition to ketamine/xylazine sedation. Eyes were disinfected with 5% povidone‐iodine and rinsed with sterile normal saline. Using all sterile procedures, a 2 mm circumferential limbal conjunctival incision was made using Westcott scissors at the incision site in the superior (for right eyes) or inferior (for left eyes) temporal quadrant after placing a lid speculum. The sclera was exposed by blunt dissection. Using a 20‐gauge V‐Lance blade, an incision was made 3.5 mm posterior to the limbus and lengthened slightly. Devices were grasped at their inactive border area using Jewelers forceps, were inserted leading with the grasped portion, and were then gently pushed from behind using a blunt device. The scleral incision was closed with 7–0 Vicryl suture. After visualizing the device by applying a macular lens to cornea with a small drop of Goniosol, 0.15 ml of 330 mg/ml cefazoline was administered into the conjunctiva using a 30‐gauge needle. A fundus photograph was taken to document the location of the device, and then the lid speculum was removed followed by topical administration of 0.3% ciprofloxacin.

#### 4.6.5 Ophthalmic exams

Eyes were examined by slit lamp biomicroscopy, fundoscopy, laser flare photometry, tonometry, color fundus photography and OCT at baseline screening, 24 hr, 72 hr, 6 days, 14 days, 21 days, 28 days, 56 days, 70 days, and 84 days post‐implantation.

Monkeys were placed in a supine position on an exam table for intraocular pressure (IOP) measurement. IOP was measured using a Tono Vet (iCare, Finland) rebound tonometer set to the dog (d) calibration setting. At each time point, three independent measurements were collected consecutively from each eye. The mean IOP value for each eye at each time point was calculated for analysis.

Anterior segment inflammation was examined with slit lamp biomicroscopy. Evaluation of posterior wall and vitreous inflammation was performed by posterior segment slit lamp exam with a 90‐diopter lens. Scoring was applied to qualitative clinical ophthalmic findings using a modified McDonald‐Shadduck scoring system.^30^ Retinal infiltrates and hemorrhage, vascular dilation, tortuosity and sheathing, and optic disc edema were also evaluated during the fundoscopy.

Laser flare photometry measures of anterior chamber inflammation were obtained at the time of ophthalmic exams using a Kowa FM‐500 laser flare photometer (Kowa Company, Tokyo Japan). At each observation point, measurements were collected until seven acceptable readings (difference between two background measurements <15%) were obtained, and the lowest and highest readings were deleted, and the mean value ± the standard deviation was calculated as specified by the manufacturer.

Fundus images were collected using a Topcon TRC‐50EX retinal camera with Canon 6D digital imaging hardware and New Vision Fundus Image Analysis System software. Pupils were dilated prior to imaging using topical 10% phenylephrine hydrochloride and 1% cyclopentolate hydrochloride ophthalmic solutions.

OCT was performed using a Heidelberg Engineering Spectralis OCT Plus system at baseline exam. Due to equipment malfunction, follow‐up exam was performed using a new Heidelberg HRA+OCT with eye tracking and HEYEX image capture and analysis software. An overall volume scan of the entire macula was performed, consisting of 49 parallel scans of 30° in the horizontal plane positioned 50 μm apart with image averaging over 49 automatic retinal tracking frames with scan grid centered on the fovea.

#### 4.6.6 Clinical observations

Clinical observations were conducted at each ophthalmic examination time point to confirm integrity of the ocular surface and normal response to mydriatics. Twice daily cage‐side observations were also performed to evaluate the general behavior and gross pathology of the eye.

## 5 CONCLUSIONS

In this report, we demonstrate an approach for methodical design and optimization of sustained‐release devices. Utilizing membranes that achieve sustained near zero‐order release, the devices were capable of releasing a therapeutically relevant protein over several months; using a PEG‐based formulation, sufficient protein stability can be achieved over the same time course. Additionally, a proof‐of‐concept in vivo study demonstrated tolerability in non‐human primates for up to 12 weeks. While the work presented here focused on research grade aflibercept as a model protein, the results are relevant to proteins in general and can be applied to a generic protein‐based device.

## Appendix

**Table A1:** Characterization of prototype devices

	Attribute	Target	Prototypes
Design parameters	Device size	1–1.5 mm D x 10 mm L	Measured surface area 79 mm^2^ (±12 mm^2^); Estimated cylindrical geometry 1.3 mm D x 10 mm L*
	Protein load	>3 mg	3.2 mg (±0.4 mg)
	PEG load	0.9 mg	0.88 mg (±0.08 mg)
	Total solid load (protein, excipients, PEG)	5 mg	5.45 mg (±0.65 mg)
	Porous area	½ of device surface area (31–47 mm^2^)	39 mm^2^ (±6 mm^2^)
System parameters	Release rate	30 μg/day	25 μg/day
	Area‐normalized Release rate	0.76 μg/(mm^2^·day)	0.64 μg/(mm^2^·day)
	PEG3350 reservoir Concentration	70 mg/ml	66 mg/ml (± 3 mg/ml)^1^
	Protein reservoir Solubility	7 mg/ml	8 mg/ml (± 1 mg/ml)
